# Ecology of *Anopheles darlingi *Root with respect to vector importance: a review

**DOI:** 10.1186/1756-3305-4-177

**Published:** 2011-09-16

**Authors:** Hélène Hiwat, Gustavo Bretas

**Affiliations:** 1 Laboratory of Entomology, Wageningen University and Research Centre, PO Box 8031, 6700 EH Wageningen, The Netherlands; 2 Malaria Program, Ministry of Health Suriname, c/o Bureau of Public Health Suriname, Rode Kruislaan 13, Paramaribo, Suriname; 3 Calle Dalia 20 los Parques, Los Ceibos, Guayaquil, Ecuador

## Abstract

*Anopheles darlingi *is one of the most important malaria vectors in the Americas. In this era of new tools and strategies for malaria and vector control it is essential to have knowledge on the ecology and behavior of vectors in order to evaluate appropriateness and impact of control measures. This paper aims to provide information on the importance, ecology and behavior of *An. darlingi*. It reviews publications that addressed ecological and behavioral aspects that are important to understand the role and importance of *An. darlingi *in the transmission of malaria throughout its area of distribution. The results show that *Anopheles darlingi *is especially important for malaria transmission in the Amazon region. Although numerous studies exist, many aspects determining the vectorial capacity of *An. darlingi*, i.e. its relation to seasons and environmental conditions, its gonotrophic cycle and longevity, and its feeding behavior and biting preferences, are still unknown. The vector shows a high degree of variability in behavioral traits. This makes it difficult to predict the impact of ongoing changes in the environment on the mosquito populations. Recent studies indicate a good ability of *An. darlingi *to adapt to environments modified by human development. This allows the vector to establish populations in areas where it previously did not exist or had been controlled to date. The behavioral variability of the vector, its adaptability, and our limited knowledge of these impede the establishment of effective control strategies. Increasing our knowledge of *An. darlingi *is necessary.

## Background

The malaria vector *Anopheles darlingi *was first described in 1926 by Root and named after Dr. Samuel Taylor Darling (1872-1925), a leading expert in tropical diseases in the early twentieth century. This mosquito species has a wide geographic distribution in South and Central America, stretching from South Mexico to North Argentina, and from the East of the Andes chain to the coast of the Atlantic Ocean [1-4]. In coastal areas its distribution is restricted by the salinity of water [2]. Seasonality in *An. darlingi *population densities depends on the type and availability of breeding sites; the species is often absent or maintains low densities in regions with a long dry season [5]. *Anopheles darlingi *has an elaborate history of vector incrimination in South America [2,3,6-8] and its presence has been associated with malaria epidemics, like the one in the Paranápanema River, Brazil, in 1950 [9].

Studies on the biology and behavior of this mosquito have been relatively few, especially when considering its important role in malaria transmission and its association with severe endemic or epidemic malaria [1-3,10-13].

The malaria situation in the world is changing. Not only is there an increased international effort to control and where possible eliminate malaria (Roll Back Malaria, Millennium Development Goals [14], supported by a changed funding environment, but also ongoing developments have resulted in new tools for malaria and vector control. Current strategies of integrated vector management, including the use of insecticide treated bed nets and indoor residual spraying, may not be sufficient to eliminate malaria transmission in all endemic areas. Considering the long-term challenge of eradication of malaria it is essential to increase knowledge on the ecology and behavior of malaria vectors like *An. darlingi*, in order to evaluate appropriateness and impact of these strategies [15,16].

### Vector importance of *An. darlingi *in the countries of distribution

**Figure 1:**
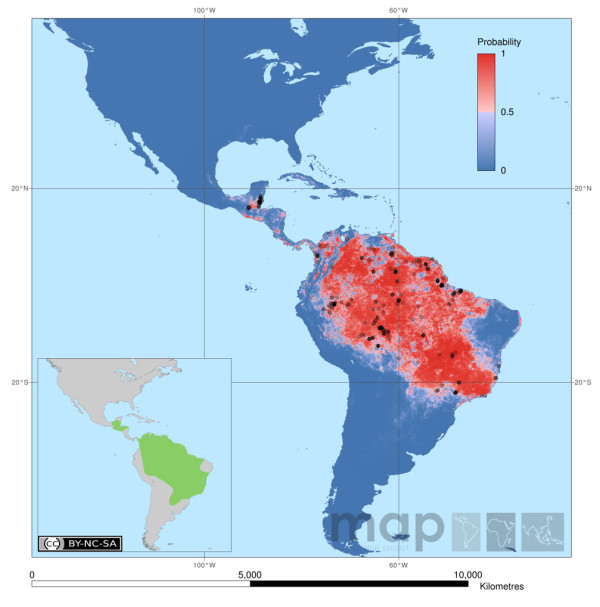
**Map of the predicted distribution of Anopheles darlingi**. Map by Sinka et al. [7] based on hybrid data (318 occurrence data plus 500 pseudo-presences weighted at half that of the occurrence data and randomly selected from within the Expert Opinion range).

Intensified malaria control activities have led to a decrease in the number of malaria cases in many countries in Latin America. According to the WHO [17], Mexico, El Salvador, Paraguay and Argentina have entered the elimination or pre-elimination phase and only four countries in the Americas, namely Brazil, Colombia, Haiti and Peru, are responsible for 90% of the malaria in this region in 2009. *Anopheles darlingi *is among the most efficient malaria vectors in the Neotropical Region [18]. The exact extent of its distribution is subject to discussion and changes continuously due to ecological changes and adaptations of this mosquito. A predicted distribution based on published records and expert opinions was recently produced by Sinka et al. [8] (Figure 1). The vector importance of *An. darlingi *varies throughout its area of distribution. In **Mexico **the main vectors in areas of high transmission risk are *Anopheles pseudopunctipennis *(inland) and *An. albimanus *(coastline and marshland). *Anopheles vestitipennis *is considered a secondary vector [19]. *Anopheles darlingi *plays a minor role in the south-eastern region of the country [20,21], especially in the state of Oaxaca. Overall malaria in Mexico is down since a change of control strategy towards ecological measures (clearing of vegetation around houses and in waterways) in 1998 [22]. In **Guatamala **the main vectors are *An. albimanus, An. pseudopunctipennis *and *An. vestipennis*, but *An. darlingi *has been collected along the various river systems [4,21,23] and is considered to play a role in the malaria transmission [24]. The same four vectors play an important role in malaria transmission in **Belize **and **Honduras **[18]. In Belize *An. darlingi *is uncommon in the northern part of the country. In the southern regions it is mostly endophagic, more so than *An. albimanus *[25]. An interesting study by Roberts et al. [26] shows that in the Toledo District, where *An. darlingi *was the most common species in the 1940s (based on Kumm & Ram [27]), no *An. darlingi *were found in 1992. Instead, the most abundant mosquito was *An. vestitipennis*. Malaria transmission in Belize decreased after 1995 as result of a vector control programme with DDT [18]. In Honduras, *An. darlingi *was a suspected vector in severe malaria transmission in the early 20^th ^century [4]. Sugar-cane and cotton farming have dried up the southern part of the country, which led to a significant decrease of malaria cases and a move of the human population to the north. The resulting forest clearing in the north, however, has led to an increase of malaria in that area due to *An. albimanus *[18,28].

The incidence of malaria in **El Salvador **is among the lowest in the Central American countries. *Anopheles darlingi *specimens have been found [21], but transmission is considered due mainly to *An. albimanus *and *An. pseudopunctipennis*, as is the case in **Nicaragua **where malaria transmission was high but is decreasing [29,30]. The northern and eastern regions still have a high transmission risk [31]. Nicaragua and **Costa Rica **have no collection data of *An. darlingi *despite numerous and comprehensive surveys in the area trying to locate it. Official reporting of *An. darlingi *from **Panama **did not exist for a long time (although it was one of the first countries worldwide from where successful malaria control was reported [32]). Malaria transmission is considered due to *An. albimanus *and *An. punctimacula*, which are the most abundant species with the highest human biting rate. *Anopheles aquasalis *is suspected of playing a role in transmission on the eastern Atlantic coast. Recent genetic studies suggest a long and stable population of *An. darlingi *in (eastern) Panama, possibly originating from Colombia [33,34]. In **Colombia**, *An. darlingi *is the principal malaria vector in the Llanos Orientale, The Amazonia, The Orinoquia, Urubai, Bajo Cauca and Magdalena Media [35]. The most important vector in Colombia is *An. albimanus*, representing 99% of the *Anopheles *population along the Pacific coast and 61% of the population along the Atlantic coast [36]. **Venezuela **has a very diverse *Anopheles *population. *Anopheles darlingi *is especially important in the Amazonian south along the rivers [6,37,38]. Studies by Moreno et al. [13,39] showed that *An. darlingi *represented over 30% of the *Anopheles *populations in locations in the Amazonias and Bolivar state. Other important vectors are *An. aquasalis *(north east coast), *An. albimanus *(coast and subcoastal areas), *An. pseudopunctipennis *(Andes foothills) and *An. nuneztovari *(northwest) [18]. Vector control with DDT between the 1940s and 1960s proved very efficient in reducing malaria in the **Guianas **(Guyana, Suriname, French Guiana), eliminating it from the coastal areas of Guyana (1951) and Suriname (1968) [37,40,41]. *Anopheles darlingi *is considered the most important and often only malaria vector in the sparsely populated interiors of the three countries which are covered with rainforest [6,39,41-45]. Hudson [46], for instance, found that females of *An. darlingi *were the commonest (98.6%) of the 5,464 anophelines he collected in the rainforest of Suriname during 1979-1981. In 1980 malaria re-appeared in the coastal area of Guyana and transmission continues among the inland Amerindians [18,40]. Despite the resurgence of malaria in the early 1990s and the continued high transmission among Amerindian and Maroon populations of the interior, the coast of Suriname is currently still free of malaria. Since 2005 a sharp decline in number of malaria cases is reported following a scale-up of intervention activities [17]. In French Guiana malaria continues to be a problem in the Amerindian populations along the Oyapock and Lawa Rivers and the Maroon population along the Marowijne River. Coastal malaria has disappeared except from import cases due to Haitian and Brazilian immigrants. This malaria is transmitted by *An. darlingi*, which is most likely breeding in coastal swamp areas [18,47]. Almost all cases in **Brazil **originate from the Brazilian Amazon, with 74% of the cases coming from the three states Rondonia, Pará and Mato Grosso [18]. *Anopheles darlingi *is one of the most imporant vectors, with a natural infection rate varying between 2.7% and 4.2% in the state of Pará [48]. Other vectors include *An. nuneztovari, An. triannulatus, An. oswaldoi, An. albitarsis *and *An. intermedius*. Povoa et al. [49] reconfirmed the importance of *An. darlingi *in malaria transmission in the savannah eco-region of northern Amazonian Brazil. Here, the species was named an important vector in peri-urban environments.

In **Equador **fifty percent of the human population lives in malaria endemic areas. Especially in the Amazonian plains, east of the Andes, *An. darlingi *plays an important role. By contrast, *An. albimanus *is the main vector along the Pacific coast and *An. pseudopunctipennis *transmits malaria along the slopes and southern valleys of the Andes [18]. The resurgence of malaria in **Peru **after 1991 was thought to be associated with the spread of *An. darlingi *into new areas of the Amazon Basin [11,50,51]. A study by Schoeler et al. [50] shows that in the departments of Loreto and Ucayali, where over 60,000 mosquitoes were collected, 71% of the mosquitoes were *An. benarrochi *and 24% were *An. darlingi*. The latter species was found in almost 50% of the study areas including areas where the species had not been reported before. Flores-Mendoza et al. [12] found positive specimens of both *An. benarrochi *and *An. darlingi *at rates of 0.14% and 0.98%, respectively. In **Bolivia**, *An. darlingi *is thought to have played an important role in malaria epidemics in the first half of the 20^th ^century [18]. The species is found in the plains of northeastern Bolivia in the Departments of Beni, Panel and Santa Cruz [52]. *Anopheles pseudopunctipennis *is an important vector in areas above 500 m. asl. Other vectors include *An. nuneztovari, An. triannulatus, An. marajoara *and *An. braziliensis*. **Paraguay **is a transition area between the Andean countries and Brazil. Areas of moderate malaria risk are found in the Alto Paraná, Caaguazú and Canendiyú, where *P. vivax *is transmitted [31]. Negligible risk exists in the remaining areas. *Anopheles darlingi *is the only malaria vector reported [18] and its reappearance along the Paraguay border with **Argentina **is assumed to have been the reason for an increase in malaria on the Argentinean side [53], together with increased border traffic along the Bolivian and Paraguayan border and the ecological changes due to the construction of dams in the Paraná basin. Malaria in the north-western area of Argentina (Satla and Jujuy provinces) is mostly due to *An. pseudopunctipennis *[54-56]. In the northeastern part (Missiones) *An. darlingi *is a vector [18].

From the combined studies reviewed above, it is apparent that *An. darlingi *has a very wide distribution and acts as a malaria vector in almost all South American countries, being the principle vector in the Amazon basin.

### Seasonality

Seasonality patterns of *An. darlingi *are closely related to the annual cycle of rainfall, although the relation of the occurrence of peak abundances to rainfall patterns seems to differ at different localities or regions. Deane et al. [2] discusses the species' sensitivity to dry season conditions. Rains are thought to increase availability of breeding sites [57] and peak abundances of *An. darlingi *in the rainy season have been reported [10,58-60]. The local distribution area of *An. darlingi *may expand during the rainy season, as was observed by Roberts et al. [25] in Belize who found adult females within the range of rivers all year round, but further away from rivers only in the wet season. Forattini [1] found a greater density of *An. darlingi *during the hot months in southern Brazil and considered that the annual cycle of activity of this species may depend on exogenic factors, including those which can affect the productivity of breeding sites. He considered that the occurrence of heavy rains could possibly flood breeding places and create flood currents that carry away immature forms. This was also found by Pajot et al. [42] in French Guiana who discovered that heavy rains are followed by a decrease in and sometimes a total absence of *An. darlingi*. This mechanism is thought to have some influence in the increase in the density of *An. darlingi *in the dry season or in the transitional period between the dry and the wet season [1,61]. Following a study in Rondônia (Brazil), Gil et al. [62] make a distinction between malaria of riverine areas and dry land malaria, explaining that differences in the nature and timing of establishment of breeding sites favorable to *An. darlingi *may result in different ecological models for malaria transmission. In dry land conditions, rivers can flood the margins during the rainy season, but the strong water flow will prevent successful breeding. Only after rains have ended, when rivers retreat to their original size, breeding sites originate as large stable water collections left behind in previously inundated areas. This results in high mosquito densities at the beginning of the dry season and malaria outbreaks in the dry season. By contrast, in riverine areas water levels of the major rivers rise significantly during the rainy season, due to draining from the tributaries. Not only the river margins, but complete forest areas can be flooded, creating inundated forest floors with low water flow which serve as excellent breeding sites for *An. darlingi*. Peak occurrences will be found in the rainy season, decreasing at the beginning of the dry season, when the flooded forest floors dry up. Such differences in ecological settings may explain a study in Suriname, where Hudson [46] discovered two different seasonal patterns (rainy season peak density and dry season peak density) in areas only 40 km. distant. Rozendaal [63] adds to the evident diversity of seasonal patterns when he finds that peaks in biting densities in a focus of malaria along the Marowijne River in Suriname correlated well with periods of (i) high water level in the long rainy season, (ii) low water level in the long dry season, and (iii) abundant rainfall in the short rainy season. Critical levels of river height and rainfall were defined, which could explain most of the monthly fluctuations in malaria parasite incidence observed in this area. Knowledge about the relationship of *An. darlingi *with environmental conditions and the impact of seasonal cycles on the mosquito population densities is required to predict areas and seasons of high malaria risk. Considering the large variety of larval habitats of *An. darlingi*, depending on the (changes in) ecological environments, and considering the adaptability of the vector, there is no way of assessing seasonality of the population densities than through the study of local settings.

### Larval habitats

Larvae of *An. darlingi *are thought to require a stable chemical and physical condition in the breeding sites, which is generally not found in small water bodies [2]. This species breeds preferentially in large, deep and clear water collections like lakes, swamps or large rivers [2,3,10,63]. Adequate larval conditions depend on depth of the water, temperature, pH, chemical stability and light/shade proportions [3]. A combination of shade and direct sunlight, with a certain amount of cover in the form of vertical vegetation is preferred, with a temperature of 20-28°C, and a pH 6.5-7.3 [3,57]. Singer and Castro [64] considered the forest margins to be the principal breeding sites for *An. darlingi *in the Amazon. Undisturbed forests rarely provide ideal breeding sites due to a high acidity of the water and an absence of partly shaded water bodies. Black rivers with a pH below 5 and with little vegetation or plankton are unsuitable breeding sites, but Giglioli [57] found how intense rainfall created adequate breeding conditions by diminishing the acidity of black water rivers and by creating clear water swamps in areas where the water bodies had a low pH or high salinity. Rozendaal [41] collected *An. darlingi *larvae in water from at a temperature of 40°C, which contradicts the consideration that the breeding sites are always in cool waters [2]. This has implications for the range of possible breeding sites for this species, especially when considering ecological changes due to for instance deforestation, dam construction or mining. Vittor et al. [65] found that mosquito breeding sites with *An. darlingi *larvae have an average of 24% forest cover, compared with 41% for sites without *A. darlingi*. Further analysis of breeding-site characteristics identified seasonality, presence of algae, size of water body, presence of human populations, and the amount of forest and secondary growth as significant determinants of *A. darlingi *presence. Larvae are generally found around trunk, emergent plants and floating debris [7,46,63,66], which seems to provide them with some amount of shadow and a stability of water condition in terms of water flow in this particular microhabitat.

Table 1 provides an overview of the various breeding sites of *An. darlingi*. When breeding in the large sites, *An. darlingi *larvae seem to prefer deeper areas, away from the edges. This is an important consideration as it causes difficulty for larval surveillance, which may lead to biased information on larval presence and population densities.

**Table 1:** Categorical overview of breeding sites of *An.darlingi*

Breeding site category	Breeding site characteristics	References
Lakes and Lagoons	in lake side vegetation and floating debris, in more exposed deeper parts of the lakes	[2,3,7,10,57,91,128,142]
Large rivers	Semi-shaded, along the river edges, between floating debris and superficial vegetation, sections with slow water flow	[2,3,9,21,42,63,66]
Small rivers	Slow flow rivers, creeks, residual pools in riverbeds during the dry season, irrigation canals	[7,46,57,101,143]
Flooded forest	Flooded forested riversides in the rainy season, swamps	[2,7,57,63,91,144]
Small water collections	Ditches, drains, wells, rain pools, old, abandoned (mining) pits	[2,3,10,57,145]
Rare breeding sites	Fully shaded water bodies, very small water collections, brackish or acid water, polluted water	[2,3,21,64,71]

### Vectorial Capacity

According to Dean [2]*An. darlingi *is capable of maintaining a relatively high transmission of malaria even when found in low densities. The mosquito species is considered a good vector, despite its infection rates tending to be low, even in high risk malaria areas. The re-emergence of malaria in eastern Peru is for instance attributed to the spread of *An. darlingi *into new areas, but the vector infection rate found was less than one percent [12,50]. In studies in the high-malaria risk area Rondônia in Brazil by Tadei [7] and Oliveira- Ferreira et al. [67], an infection rate in *An. darlingi *of 0.48% (one positive out of 210 and 47 positive out of 9,838, respectively) was found. Gil et al. [62] also found a low infection rate and considered that *An. darlingi*, which is the primary vector in that area, maintains malaria transmission by its high human biting rate and that transmission is supported through the high number of asymptomatic malaria cases in the human population. *Anopheles darlingi *has a relatively high susceptibility to *Plasmodium *infection when compared to other Amazonian species [68], and asymptomatic cases with very low parasitemias can be infective to *An. darlingi*, even if it is at a much lower rate than symptomatic cases [69]. The recent discovery of sub-microscopic, but highly infectious, low-density gametocytes of *Plasmodium falciparum *may be an additional explanation for the relatively continuous infections in the Amazon [70]. Mosquito infection rate can be relatively high at times [48]. Da Silva Vasconcelos et al. [71] found 8.5% of over 700 *An. darlingi *collected in Roirama (Brazil) to be infected with a *Plasmodium *parasite. This rate would be comparable to infection rates found for instance for *An. gambiae *and *An. funestus *in Tanzania (11.1% and 6.2% respectively [72]).

Gonotrophic cycle, longevity and age composition are considered important vector characteristics that are essential in determining the ability to transmit malaria. Based on the view that the gonotrophic cycle in other tropical anophelines lasts approximately three days [61,73] calculated a daily mortality of *An. darlingi *at Arapuaña, Brazil, of approximately 38%. He considered that only those females that had completed four or more cycles would be old enough to contain malaria sporozoites [74]. Of 1,596 dissected females in his study, only seven had laid eggs four times or more, resulting in a proportion of females that could be potentially infective with malaria sporozoites of 0.4%. In reality the gonotrophic cycle of *An. darlingi *may be two rather than three days [75], which means that the daily mortality at Arapuaña would actually be higher than estimated by Charlwood (approximately 51%).

Terzian and Stahler [76] considered that the male-female composition of a mosquito population may influence feeding behavior, after a laboratory study with *An. quadriannulatus *in which the virgin females never took a blood meal. This supports the hypothesis that take-off or host-seeking behavior is inhibited until a substance is transmitted to the female during mating. Recent studies on the behavior of several other anopheline species show that pre-gravid biting is common and sometimes needed before mating can occur [77,78]. Multiple blood meals within a single gonotrophic cycle appear to be less important in the life histories of neotropical Nyssorhynchus species, including *An darlingi*, compared to Afrotropical malaria vectors. One blood meal is usually enough for egg maturation [79,80].

Age of female mosquitoes is estimated from the parity rate of a population, i.e. the rate of females which have had a blood meal and have laid eggs (as determined using the ovarian tracheole dissection by Detinova [81]). The reliability of this method, especially in older females is subject to discussion [82]. Age composition of female *An. darlingi *collected in the field differs over collection time, collection seasons and locality. Higher variability in age composition may be found in the dry season, possibly related to availability of breeding sites and more stable climatic conditions [83]. Hudson [46] found that the parous rates of females he caught in Suriname from 19.00-22.00 h were 10-20% higher than those of females caught at other times of the night. The differences in age composition over season and time of day or night will need to be taken into account when collecting the mosquitoes for determination of infection rate [84]. Age composition may also depend on the distance of breeding sites to nearby human settlements. More gravid females and less nulliparous females are found close to breeding sites than in villages away from breeding sites [85].

Flight range of malaria vectors is an important determinant for their success in transmission over distance. Deane et al. [2] found breeding sites as far as 1.5 to 2 km from the sites of adult captures in the Amazon. In a study in Jardim das Copaíbas (Roirama, Brazil) Monteiro de Barros et al. [80] found that 20.3% of *An. darlingi *would fly over 500 m., 4.6% would fly over 1000 m., and less than 1% further than 1200 m. In comparison, Achee et al. [86] found recovery rates of 29%, 11.6%, 5.8% at distances of 0, 400 and 800 m., respectively, in Belize. Tadei [7] estimates a possible flight range of 5 km when going downwind, and Charlwood and Alecrim [87] in a capture-recapture study in Brazil found two *An. darlingi *females at 7.2 km. from their release site, nine days after release. Recapture rate at the release site was 12-19%. This dispersal behavior is similar to what is found in African anopheline species [88]. Due to the variability of the vectorial capacity determinants and their dependence on external factors vectors, obtaining insight into local *An. darlingi *vectorial capacity is difficult and requires a sound methodology and understanding of the variabilities.

### Relative abundance and feeding behavior

*Anopheles darlingi *is attracted to the human host [57,87,89]. Deane et al. [2] and Rachou [3] compared the attraction of the mosquitoes to humans and other mammals and found that especially humans and large mammals, like horses, are preferred. Zimmerman et al. [89] considered that host selection may in fact vary much depending on host availability and can differ significantly in different sites within one region. The tendency of *An. darlingi *to go indoors for biting was confirmed very early [2,90,91]. A study in Belize showed an indoor-outdoor ratio of 1:0.6 for *An. darlingi *[25]. In Brazil a larger degree of variation in behavior was found by Deane et al. [2], who discovered a stronger exophilic tendency in the Interior of Brazil. Charlwood [37] confirmed this with his study in Manaus, where he found *An. darlingi *to be primarily exophagic and exophilic, while in northern Brazil (towards the border with Guyana and Venezuela) it was more endophagic. Rozendaal [92] captured 73% of the *An. darlingi *in the Interior of Suriname outside of the houses, in the peridomestic areas. He discovered that, even when *An. darlingi *is anthropophilic and prospers in the presence of human blood, it is able to survive as a 'wild' population in much lower numbers on animal blood. This was also found in French Guyana by Pajot [93], and in Brazil by Deane et al. [2] where *An. darlingi *was collected in uninhabited areas.

Elliot [58] found that in periods of increased mosquito density the relative importance of outdoor biting (during times of human activity) declines. He offers two possible explanations, the first one being that in periods of highest density the females may have a 48 hr gonotrophic cycle, causing them to lay eggs in the early evening and start feeding in the late night of the same night. The second explanation would be that high density of mosquitoes often coincides with periods of heavier rainfall and higher relative humidity. Relative humidity inside houses, which may well inhibit entry when it is low in the first hours after sunset, would rise sooner in the wet season as the house cools, which may result in higher numbers of entry. Either or both hypotheses could be true. Hudson [46] studied the resting time of mosquitoes before and after biting in Suriname and found at Aselikamp in June 1979 that the mean resting periods observed were 7.7 min (range 1-35) for 52 unfed females, and 17.1 min (range 2-41) for 10 blood fed females.

Biting cycles of *An. darlingi *seem to differ significantly between various regions of its distribution area, and even in localities not far apart. Some studies record unimodal [94] and other bimodal biting rythms [1,7,42,59,95]. Da Silva-Vasconcelos [71] found no defined biting peak for *An. darlingi *in Boa Vista (Roraima, Brazil) where over 10% of the anopheline population was found to be *An. darlingi *and where *An. darlingi *had the highest *Plasmodium *infection rate and together with *An. albitarsis *was considered the prime vector for this area. Forattini [1] found that the distribution of the 9,523 *An. darlingi *caught in southern Brazil at 1500 feet displayed a distinct bimodal distribution in the daily blood feeding periodicity with peaks at both dusk and dawn. Hudson [46] discovered that biting cycles of *An. darlingi *at his two study sites (Aselikamp and Apoma Tapoe, Suriname) showed a single main peak, but the peak would occur one hr later at Aselikamp (22.00-23.00) than at Apoma Tapoe (21.00-22.00), with smaller secondary peaks at 18.00-19.00 and 5.00-6.00 hr. Pajot et al. [42] found a trimodal cycle in nearby French Guiana, including both twilight periods and a clear nocturnal peak between 1.00-2.00. He also found that cycles of biting activity of parous and nulliparous females are similar, both inside the house and on the veranda. That different biting cycles of *An. darlingi *can be found in a single locality over the seasons was discovered by Leon et al. [96] in St Clara (Peru) where a unimodal cycle was found from August to December and a bimodal cycle from January to June. Vector control activities like Indoor Residual Spraying (IRS) or the use of insecticide treaded nets (ITNs) can result in a change in biting behavior [96-98].

Lunar cycles do not appear to influence daily biting rhythms of most mosquito species, but larger numbers of mosquitoes can be collected during new moon [99]. Voorham [100] discarded the likelihood of mosquito density interfering with biting behavior after his study in the State of Amapa (Brazil), this is consistent with studies in for instance French Guiana [42], but is not in line with results obtained in studies in Colombia and Brazil [101]. Voorham acknowledges that intra-population variation of biting activity can be as significant as inter-population variation, and states that plasticity in biting activity patterns can result in increased vectorial potential of mosquitoes and control strategies may have to be adjusted to account for difference in human-vector contact over time.

Mosquitoes may very well display a preference in their biting sites on their available or preferred host. Observations by De Jong and Knols [102] on mosquito biting on humans revealed that many species have preferred biting sites, and that not all species share the same preferences. Selection of these sites may be related to several factors, depending on the mosquito species, including visual and chemical properties of the host. This was confirmed by the differential attractiveness of Kenyan men to the African malaria vector *An. gambiae *[103] and another study that revealed that allomonal breath contributes to differential attractiveness of humans to the African malaria vector *An. gambiae *[104]. In a study on the South American malaria vector *An. albimanus*, biting sites were recorded mostly from the head region, suggesting that this species responds mostly to human breath [105]. Studies on biting site preferences of *An. darlingi *are necessary to provide information on cues that are important in the finding and selection of a host. This information could be applied in trapping and control activities [106].

### Biological variation

The possibility that the mosquito species *An. darlingi *may consist of a species complex is subject of continued research. If true, it could have important implications for future malaria control schemes in Latin America. Charlwood [37] found that *An. darlingi *mosquitoes from the Manaus area are more chromosomically diverse than mosquitoes towards the northern edge of the distribution area (Venezuela and Guyana). He also found that female wing size can vary between populations. Wing size variation in mosquitoes can in fact be due to other than genetic variation, for instance to differences in larval population densities and food availability, as was shown for *Aedes aegypti *by Jirankanjanakit et al. [107]. A study by Harbach et al. [108] showed that the *An. darlingi *specimens found in Belize show variation in their hind tarsal markings at a more than incidental rate.

Rosa-Freitas et al. [109] related iso-enzymatic, behavioral and mitochondrial DNA studies on Brazilian and other Latin-American populations and deducted that *An. darlingi *is a monotypic species. Mirabello and Conn [110] studied the genetics of *An. darlingi *mosquitoes to determine whether there is a division in the gene pool between Central and South America and found no significant evidence for this. Conn et al. [111] continued this research in an attempt to find a population bottleneck in *An. darlingi *due to possible pressure as a result of insecticide use. The bottleneck was not found but significant differentiation between locations north and south of the Amazon River were discovered, suggesting a degree of genetic isolation between them, which was attributed to isolation by distance. Continued studies by Mirabello et al. [112] result in the conclusion that "all of the data confirm a deep divergence between Amazonia and southern Brazil (genotype 1), and Central America, Colombia, and Venezuela (genotype 2).", which indicate incipient speciation. Recent studies in Brazil and Colombia show that on a more local level speciation is less likely due to high levels of gene flow, although even on that level evidence for isolation by distance exists [113,114].

### Ecological change

In the whole of South America ongoing development results in changing environments: agriculture and industries, colonization of uninhabited areas by humans, construction of hydropower dams, and forestry and mining activities are some of the causes. The globally changing climate is another. Change of ecosystems can result in a change in availability of breeding sites for mosquitoes or a change of survival rate and reproduction [115,116]. This may affect the malaria transmission risk. According to Patz & Olson [117], changing temperature trends, due to influences from global climate change and local land use practices, may alter malaria risk, due to 1) a shift in time needed for parasite development, 2) changing mosquito abundance and survivorship, 3) a change in gonotrophic cycle, and 4) a change in larval development and pupation rates. Non-sustainable forestry, resulting in large-scale deforestation, will have an effect on local temperature, and possibly on the availability of breeding sites. Vittor [118] found that the biting rate of *An. darlingi *is positively related to the amount of deforested land, and further found that deforested sites had an *Anopheles darlingi *biting rate that was more than 278 times higher than the rate determined for areas predominantly forested [119]. In accordance, Harris et al. [120] considered that the growing malaria problem in the Bolivian Amazon (a four-fold increase between 1991 and 1998) was largely due to forest clearance, bringing human and vector populations into closer contact. Malaria outbreaks were predicted for Belem (Brazil) as a result of the continued expansion of the city into the surrounding forest in the 1990s, and the observed increase in the population sizes of *An. darlingi *in these locations [121].

The construction of hydropower dams often came with special awareness of a possible increase of the malaria risk, which resulted in related studies. De Carvallo [122] found a decline in *An. darlingi *densities and malaria transmission after the construction of the Lages dam in Brazil. This was attributed to the variations in water level, which destroyed the preferential breeding places of *An. darlingi*. Rozendaal [63] found that, in contrast to the prediction by Van Thiel [123], the hydropower scheme which created Van Blommenstein lake in 1971 in Suriname, did not cause a malaria problem. No *An. darlingi *were found in that area. He assumed that the non-shaded shores of the lake are unsuitable breeding habitats. With the completion of the (binational) Itaipu dam between Brazil and Paraguay and the maintenance of the breeding places for *An. darlingi *an increasing number of malaria cases, especially in the Upper Parana River, was expected. Falavigna-Guilherme et al. [9] describe the occurrence of some *P. vivax *outbreaks after the completion of the dam, but believe that with the adoption of satisfactory preventive measures, including health educational and social actions, malaria can be controlled. Zeilhofer et al. [124] found a positive relationship between the *An. darlingi *presence and increased proximity to forested areas near reservoirs, especially in bays protected from wind and wave action. Breeding site classification with satellite imaging together with entomological studies are proposed as a valuable tool for spatial modeling of *An. darlingi *habitats in hydropower reservoir areas [124].

One increasingly common human activity in South America is gold mining, especially in the forested areas of the Amazon. The relation between gold mines and malaria has been discussed often [125]. In fact, the re-emergence of *P. falciparum *malaria in Cuyuni-Mazaruni-Potaro in Guyana, after 28 years of absence of cases, was considered due to the 'gold rush' [126]. In Mato Gross (Brazil) a positive correlation existed between amount of gold extraction and malaria incidence rate [127]. Very early on it was recognized that in mining areas, old abandoned pits could be suitable as *Anopheles *breeding sites [57] and this was recently re-established [13]. Clear and deep water bodies remain after sand and debris are deposited on the bottom of the mining pits over time. Destruction of the surrounding forest by the mining activities may result in less than optimal amount of shadow, but *An. darlingi *has shown tolerance to high water temperatures [41] and an ability to adapt. Following a successful malaria intervention program in the Interior of Suriname between 2005 and 2010, the only persistent malaria areas are associated with gold mines (H. Hiwat, unpublished results).

### Surveillance and Control

Capturing *An. darlingi *is not easy. So far, human landing catches seem the 'golden standard' for collecting this vector. The generally disappointing results of various trapping devises when compared to human landing collections (see for instance Moreno et al. [39], Brochero et al. [128], Turrel et al. [129], Dusfour et al. [130]) may lie in the high degree of anthropophily which is often found in this species. This high degree of anthropophily could indicate that *An. darlingi *is attracted by very specific human-related cues, and is therefore less inclined to enter traps which fail to present these cues. Since human landing catches are costly and labor-intensive and may present a risk to the collector, alternative methods are needed. Further studies into the biting behavior and preferences of *An. darlingi *may be instrumental in the development of an efficient alternative collecting method which can be used in vector surveillance. Identifying the host-related cues which attract the vector can ultimately result in more target-specific control measures. This line of study and tool development is currently employed for *An. gambiae *s.s. surveillance and control [131].

Evidence exists that vector control measures can result in changing characteristics of the targeted vector [132]. Long-term DDT use resulted in a changed susceptibility of *An. darlingi *populations over time [133]. Indoor Residual Spraying (IRS) and Insecticide Treated Nets (ITNs) reduced intra-domiciliary vector densities in several species and variation in biting time after their introduction has been recorded [134,135]. Which vector control method to use at a certain location, depends very much on the characteristics of the vector and requires adequate baseline information and continuous monitoring to detect changes [136]. ITNs for instance proved very successful against *An. darlingi *in southern Venezuela, where a reduction of 56% of malaria cases was recorded in local indigenous populations after the introduction of lambdacyhalotrin-treated hammock nets [137]. The use of same ITNs in the Bolivian Amazon might be less successful because, as Harris et al. [120] found, 48% of mosquito biting takes place between 19.00 and 21.00 h., when most people are not yet in the protective area of the net.

The mobility of larval mosquitoes is low compared to that of the adult forms, which is why larval control of vectors can be a powerful tool in malaria control [138]. Possible options are biological control with larvivorous fish or bacteria, or chemical control, for instance with oil. Whether larval control can be successful depends on the characteristics, especially the size, of breeding habitats [139]. *Anopheles darlingi *breeds in various habitats, smaller and larger, often covering wide areas (see above). In clearly defined breeding sites, like the stagnant water bodies remaining after the rainy season, limited areas of swamps or inundated forest floors, and possibly mining pits or other limited breeding sites derived from human activity, larval control with microbial larvicides could be effective. Bacterial control has been successful in Africa when directed at *An. gambiae *[140], but also in breeding sites in Peru and Equador [141]. The difficulty of localizing the breeding sites where larval control of *An. darlingi *could be successful to reduce malaria incidence is yet another issue. The Amazon rainforest is still a sparsely inhabited area, and the logistics of locating and treating individual breeding sites may preclude control directed at the larval stage. Satellite imaging may prove a useful tool in this endeavor. Studies on this subject are currently undertaken [124].

## Conclusions

*Anopheles darlingi *is widely distributed across South America, but the species is especially important as malaria vector in the Amazonian countries. Even though the natural infection rate and population densities of this vector are often low, its efficiency in malaria transmission, through high biting rates and a good susceptibility to *Plasmodium *infection, is high. *Anopheles darlingi *is mostly anthropophilic and shows a capacity to adapt to changing environmental situations. Local variability in determinants for the vectorial capacity of *An. darlingi *is high and many aspects determining this capacity, i.e. its relation to seasons and environmental conditions, its gonotrophic cycle and longevity, and its feeding behavior and biting preferences, are still unknown. This means that the establishment of an effective control strategy will require elaborate studies on the (local) vector situation. Also this behavioral plasticity makes it difficult to predict the impact of changes in ecological environment and in (macro) climate on the vector populations. Adaptation through natural selection is to be expected. This allows the vector to establish populations in areas where it previously did not exist or had been controlled. The behavioral variability of the vector, its adaptability, and our limited knowledge of it impede the establishment of effective control strategies. Increasing our knowledge of *An. darlingi*, therefore, is necessary.

## Competing interests

The authors declare that they have no competing interests.

## Authors' contributions

HH conceived and wrote the article. GB helped with its design and content. Both authors read and approved the final manuscript.

## References

[B1] Forattini OP Compartemento exofilo de *Anopheles darlingi *Root, em regiảo meridional do Brasil. Exophilic Behavior of Anopheles darlingi Root in a southern region of Brazil Rev Saứde Publ 1987 21 291 304 3445112 10.1590/s0034-89101987000400002

[B2] Deane LM Causey OR Deane MP Notas sôbre a distribuição e a biologia dos anofelinos das regiões nordestina e amazônica do Brasil Rev Serv Espec Saude Publ 1948 1 827 965

[B3] Rachou RG Anofelinos do Brasil. Comportamento das species vetoras de malaria Rev Braz Malariol Doenças Trop 1958 10 145 181

[B4] Komp WHW The occurrence of *Anopheles darlingi *Root in Central America Am J Trop Med Hyg 1941 21 659 670

[B5] Moutinho PR Gil LHS Cruz RB Ribolla PEM Population dynamics, structure and behavior of *Anopheles darlingi *in a rural settlement in the Amazon rainforest of Acre, Brazil Malar J 2011 10 174 10.1186/1475-2875-10-174 21702964 PMC3146439

[B6] Giglioni G Biological variations in *Anopheles darlingi *and *Anopheles gambiae*. Their effect on practical malaria control in the Neotropical Region Bull World Health Org 1956 15 461 471 13404433 PMC2538274

[B7] Tadei WP Santos JMM Costa WLD Scarpassa VM Biologia dos Anopfelinos Amazônicos, Occorência de Espécies de Anopheles, Dinâmica da Transmissão e Controle da Malária na Zona urbana de Ariquemes (Rondônia) Ver Inst Med Trop Sâo Paolo 1988 30 221 251 10.1590/s0036-46651988000300017 3065910

[B8] Sinka ME Rubio-Palis Y Manguin S Patil AP Temperley WH Gething PW Van Boeckel T Kabaria CW Harbach RE Hay SI The dominant *Anopheles *vectors of human malaria in the Americas: occurrence data, distribution maps and bionomic Precis Parasites and Vectors 2010 3 72 10.1186/1756-3305-3-72 20712879 PMC2936890

[B9] Falavigna_Guilherme AL Da Silva AM Guilherme EV Morais DL Retrospective study of malaria prevalence and *Anopheles *genus in the area of influence of the binational itaipu reservoir Rev Inst Med Trop S Paulo 2005 47 81 87 10.1590/S0036-46652005000200004 15880218

[B10] Forattini OP Entomología médica, vol I. Parte Geral, Diptera, Anophelini Sao Paolo, Faculdade de Higiene e Saude Publica 1962 Universidade de Sao Paolo

[B11] Aramburú J Ramal C Witzig R Malaria Reemergence in the Peruvian Amazon Region Emerg Infect Dis 1999 5 209 215 10.3201/eid0502.9902 10221872 PMC2640690

[B12] Flores-Mendoza C Fernández R Escobedo-Vargas KS Vela-Perez Q Schoeler GB Natural *Plasmodium *Infections in *Anopheles darlingi *and *Anopheles benarrochi *(Diptera: Culicidae) from Eastern Peru J Med Entomol 2004 41 489 494 10.1603/0022-2585-41.3.489 15185955

[B13] Moreno JE Rubio-Palis Y Páez E Pérez E Sánchez V Abundance, biting behavior and parous rate of anopheline mosquito species in relation to malaria incidence in gold mining areas of Southern Venezuala Med Vet Entomol 2007 21 339 349 10.1111/j.1365-2915.2007.00704.x 18092972

[B14] United Nations We can end poverty 2015: Millennium Development Goals 2010 http://www.un.org/millenniumgoals/

[B15] Ferguson HM Dornhaus A Beeche A Borgemeister C Gottlieb M Mulla MS Gimnig JE Fish D Killeen GF Ecology: A prerequisite for Malaria Elimination and Eradication PLoS Med 2010 7 8 e1000303 10.1371/journal.pmed.1000303 20689800 PMC2914634

[B16] MalERA Consultative Group on Vector Control A Research Agenda for Malaria Eradication: Vector Control PLoS Med 2011 8 1 e1000401 21311587 10.1371/journal.pmed.1000401 PMC3026704

[B17] World Health Organization World Malaria Report 2010 2010 http://www.Who.int/malaria/world_malaria_report_2010/en/index.html

[B18] Manguin S Carnevale P Mouchet J Coosemans M Julvez J Richard-Lenoble D Sircoulon J Biodiversity of malaria in the world Montrouge, France, John Libbey Eurotext 2008

[B19] Santamarina Mijares A Pérez Pacheco R Tomás Martínez SH Enrique Cantón L Flores Ambrosio G The Romanomermis iyengari parasite for Anopheles pseudopunctipennis suppression in natural habitats in Oaxaca State, Mexico Rev Panam Salud Publica 1999 5 23 28 10.1590/S1020-49891999000100004 10050611

[B20] Loyola EG Arrendondo JI Rodriguez MH Browns DN Vasa Marin MA *Anopheles vestitipennis*, the probable vector of *Plasmodium vivax *in the Lacandon forest of Chiapas, Mexico Trans Roy Soc Trop Med Hyg 1991 85 171 174 10.1016/0035-9203(91)90010-V 1887463

[B21] Manguin S Roberts DR Andre RG Rejmankova E Hakre S Characterization of *Anopheles darlingi *(Diptera: Culicidae) larval habitats in Belize J Med Entomol 1996 33 205 211 8742522 10.1093/jmedent/33.2.205

[B22] Shah S The battle against malaria; turning away from DDT to greener weapons http://www.worldchanging.com/archives/011134.html

[B23] Mirabello L Conn JE Molecular population genetics of the malaria vector *Anopheles darlingi *in Central and South America Heredity 2006 96 311 321 10.1038/sj.hdy.6800805 16508661

[B24] Hernandez MM Recinos MG Salazar V Flores R Rodas A Variacion Metrica inter e intrasepcifica en mosquitos del genero Anopheles vectores de la malaria en Guatamala San Carlos, Direccion General de Investigation Universidad de San Carlos de Guatamala 2009

[B25] Roberts DR Manguin S Rejmankova E Andre R Harbach RE Vanzie E Hakre S Polanco J Spatial distribution of adult *Anopheles darlingi *and *Anopheles albimanus *in relation to riparian habitats in Belize, Central America J Vector Ecol 2002 27 21 30 12125869

[B26] Roberts DR Chan O Pecor J Rejmankova E Manguin S Polanco J Legers J Preliminary observations on the changing roles of malaria vectors in southern Belize J Am Mosq Contr Assoc 1993 9 456 459 8126482

[B27] Kumm HW Ram LM Observations on the *Anopheles *of British Honduras Am J Trop Med Hyg 1941 21 Suppl 1 559 566

[B28] Almendares J Sierra M Anderson PK Epstein PR Critical conditions; a profile of Honduras The Lancet 1993 342 1400 1402 10.1016/0140-6736(93)92758-L 7901687

[B29] Garfield R Malaria control in Nicaragua: social and political influences on disease transmission and control activities The Lancet 1999 354 414 418 10.1016/S0140-6736(99)02226-6 10437886

[B30] PATH Malaria in Nicaragua. A review of control status trends and needs 2010 http://www.path.org/files/TS_nicaragua_malaria_rpt.pdf

[B31] Centers for Disease Control and Prevention Health Information of International Travel 2010 http://wwwnc.cdc.gov/travel/content/yellowbook/home-2010.aspx

[B32] Dehné EJ Fifty years of malaria control in the Panama area Am J Trop Med Hyg 1955 4 800 811 13259004

[B33] Loaiza JR Bermingham E Scott ME Rovira JR Conn JE Species Composition and distribution of adult *Anopheles *(Diptera; Culicidae) in Panama J Med Entomol 2008 45 841 851 10.1603/0022-2585(2008)45[841:SCADOA]2.0.CO;2 18826025 PMC2874935

[B34] Loaiza JR Scott M Bermingham E Rovira JR Sanjur O Conn JE Short Report: Anopheles darlingi (Diptera: Culicidae) in Panama Am J Trop Med Hyg 2009 81 23 26 19556561 PMC2769027

[B35] Olana VA Brochero H Saenz R Quinones ML Molina JA Mapas preliminaries de la distribution de species de anopheles vectores de malaria en Colombia Biomedica 2001 21 402 408

[B36] Guttierez LA Naranjo N Jaramillo LM Muskus C Luckhart S Conn JE Correa MM Natural Infectivity of *Anopheles *species from the pacific and Atlantic regions of Colombia Acta Trop 2008 107 99 105 10.1016/j.actatropica.2008.04.019 18554564

[B37] Charlwood JD Biological variation in *Anopheles darlingi *Root Mem Inst Oswaldo Cruz 1996 91 391 398 9070397 10.1590/s0074-02761996000400001

[B38] Magris M Rubio-Palis Y Menares C Villegas L Vector bionomics and malaria transmission in the Upper Orinoco River, Southern Venezuela Mem Inst Oswaldo Cruz 2007 102 303 311 10.1590/S0074-02762007005000049 17568935

[B39] Moreno J Rubio-Palis Y Pérez E Sánchaes V Páez E Evaluación de tres métodos de captura de anofelinos en un área endémica de malaria del estado Bolivar, Venezuela Entomotropica 2002 17 157 165

[B40] Rambajan I Re-appearance of *Anopheles darlingi *Root 1926 and vivax malaria in a controlled area of Guyana, South America Trop Geogr Med 1984 36 61 66 6375050

[B41] Rozendaal JA Observations on the biology and behavior of anophelines in the Surinam rainforest Cah ORSTROM ser Ent Méd et Parasitol 1987 15 33 43

[B42] Pajot F-X Le Pont F Molez J-F Degallier N Aggressivité d' *Anopheles *(Nyssorhynchus) *darlingi *Root, 1926 (Diptera, Culicidae) en Guyane française Cah ORSTOM ser Ent Méd et Parasitol 1977 15 15 22

[B43] Juminer B Robin Y Pajot FX Eutrope R Physionomie du paludisme en Guyane Med Trop 1981 41 135 146 7017337

[B44] Girod R Gaborit P Carinic R Issaly J Fouque F *Anopheles darlingi *bionomics and transmission of *Plasmodium falciparum, Plasmodium vivax *and *Plasmodium malariae *in Amerindian villages of the Upper Maroni Amazonian forest, French Guiana Mem Inst Oswaldo Cruz 2008 103 702 710 10.1590/S0074-02762008000700013 19057822

[B45] Hiwat H Issaly J Gaborit P Somai A Samjhawan A Sardjoe P Soekhoe T Girod R Behavioral heterogeneity of *Anopheles darlingi *(Diptera: Culicidae) and malaria transmission dynamics along the Maroni River, Suriname, French Guiana Trans R Soc Trop Med Hyg 2009 104 207 213 19732925 10.1016/j.trstmh.2009.07.007

[B46] Hudson JE *Anopheles darlingi *Root (Diptera:Culicidae) in the Surinam rain forest Bull Ent Res 1984 74 129 142 10.1017/S0007485300010002

[B47] Claustre J Venturin C Nadiré M Fauran P Malarial vectors in French Guiana: study in an epidemic focus near Cayenne (1989-1998) Bull Soc Pathol Exot 2001 94 353 357 11845534

[B48] De Arruda M Carvallo MB Nussenzweig RS Maracic M Ferreira AW Cochrane AH Potential vectors of malaria and their different susceptibility to *Plasmodium falciparum *and *Plasmodium vivax *in northern Brasil identified by immunoassay Am J Trop Med Hyg 1986 35 873 881 3532844 10.4269/ajtmh.1986.35.873

[B49] Póvoa MM Lessa de Souza RT Da Luz Lacerda RN Santa Rosa E Galiza D De Souza JR Wirtz R Schlichting CD Conn JE The importance of Anopheles albitarsis E and *An. darlingi *in human malaria transmission in Boa Vista, State of Roirama, Brazil Mem Inst Oswaldo Cruz 2006 101 162 168 10.1590/s0074-02762006000200008 16830709

[B50] Schoeler GB Flores-Mendoza C Fernández R Reyes Davila J Zyzak M Geographical distribution of *Anopheles darlingi *in the Amazon Basin Region of Peru J Am Mosq Control Assoc 2003 19 286 296 14710728

[B51] Pinedo-Cancino V Sheen P Tarazona-Santos E Oswald WE Jeri C Vittor AY Patz JA Gilman RH Limited diversity of *Anopheles darlingi *in the Peruvian AmazonRegion of Iquitos Am J Trop Med Hyg 2006 75 238 245 16896125 PMC1559519

[B52] Walter Reed Army Medical Center Disease Vector Ecology Profile Bolivia 1998 Defense Pest Management Information Analysis Center, Washington DC

[B53] De Casa C Isabel S Malaria re-infestation on the northern border of Argentina Geo Journal 1992 26 65 67

[B54] Davis NC *Anopheles pseudopunctipennis *as a malaria transmitter in Northern Argentine Republic Am J Trop Med Hyg 1927 7 167 176

[B55] Juri MJD Zaidenberg M Almiron W Distribución espacial de *Anopheles pseudopunctipennis *en las Yungas de Salta Argentina Rev Saude Publ 2005 39 565 570 10.1590/s0034-89102005000400008 16113905

[B56] Juri MJD Zaidenberg M Claps GL Santana M Almiron W Malaria transmission in two localities in northwestern Argentina Malar J 2009 8 18 10.1186/1475-2875-8-18 19152707 PMC2644309

[B57] Giglioli G Malaria in British Guiana; part III. Breeding habits of *An. darlingi *natural factors which limit the distribution of this species and of malaria Agr J British Guiana 1938 9 197 206

[B58] Elliot R Studies on Man-Vector contact in some malarious areas in Colombia Bull World Health Org 1968 31 239 253 PMC2554320 5302300

[B59] Tineo VE Medina CA Fallaque FC Chávez L Quispe S Mercado M Zevallos J León W Palomino M Distribución geográfica y comportamiento estacional de la picadura del *Anopheles *(Nyssorhynchus) *darlingi *Root 1926 en localidades de la frontera Perú-Bolivia, Madre de Dios, Perú Rev Perú Med Exp Salud Publica 2003 20 78 8321896075

[B60] León W Valle J Naupay R Tineo E Rosas A Palomino M Comportamiento estacional del *Anopheles *(Nyssorhynchus) *darlingi *Root 1962 en localidades de Loreto y Madre de Dios, Peru 1999-2000 Rev Peru Med Exp Salud Publica 2003 20 22 27

[B61] Charlwood JD Observations on the bionomics of *Anopheles darlingi *Root (Diptera; Culicidae) from Brazil Bull Ent Res 1980 70 685 692 10.1017/S0007485300007975

[B62] Gil LHS Tada MS Katsuragawa TH Ribolla PEM Pereira da Silva LH Urban and suburban malaria in Rondônia (Brazilian Western Amazon) II: perennial transmissions with high anopheline densities are associated with human environmental changes Mem Inst Oswaldo Cruz 2007 102 271 276 10.1590/S0074-02762007005000013 17568931

[B63] Rozendaal JA Relations between *Anopheles darlingi *breeding habitats, rainfall, river level and malaria transmission rates in the rain forest of Suriname Med Vet Ent 1992 6 16 22 10.1111/j.1365-2915.1992.tb00029.x 1600221

[B64] Singer BH Castro MC Agricultural colonization and malaria on the Amazon Frontier Ann N Y Acad Sci 2001 954 184 222 11797857 10.1111/j.1749-6632.2001.tb02753.x

[B65] Vittor AY Pan W Gilman RH Tielsch J Glass G Shields T Sánchez-Lozano W Pinedo VV Salas-Cobos E Flores S Patz JA Linking deforestation to malaria in the Amazon: characterization of the breeding habitat of the principal malaria vector, *Anopheles darlingi* Am J Trop Med Hyg 2009 81 5 12 19556558 PMC3757555

[B66] Achee NL Grieco JP Masuoka P Andre RG Roberts DR Thomas J Briceno I King R Rejmankova E Use of remote sensing and geographic information systems to predict locations of *Anopheles darlingi*-positive breeding sites within the Sibun River in Belize, Central America Med Entomol 2006 43 382 92 10.1603/0022-2585(2006)043[0382:UORSAG]2.0.CO;2 16619625

[B67] Oliveira-Ferriera J de Lourenco de Oliveria R Teva A Dean LM Daniel-Ribeiro CT Natural Malaria infections in anophelines in Rondonia state, Brazilian Amazon Am J Trop Med Hyg 1990 43 6 10 2200290

[B68] Klein TA Lima JBP Tada MS Comparative Susceptibility of Anopheline Mosquitoes to *Plasmodium falciparum *in Rondonia, Brazil Am J Trop Med Hyg 1991 44 598 603 1858963 10.4269/ajtmh.1991.44.598

[B69] Alves FP Gil LHS Marrelli MT Ribolla PEM Camargo EP Da Silva LHP Asymptomatic Carriers of *Plasmodium *spp. as Infection Source for Malaria Vector Mosquitoes in the Brazilian Amazon J Med Entomol 2005 42 777 779 10.1603/0022-2585(2005)042[0777:ACOPSA]2.0.CO;2 16363160

[B70] Schneider P Bousema T Gouagna LC Otieno S Van de Vegte-Bolmer M Omar SA Sauerwein RW Submicroscopic *Plasmodium falciparum *gametocyte densities frequently result in mosquito infection Am J Trop Med Hyg 2007 76 470 474 17360869

[B71] Da Silva-Vasconcelos A Neves Kató MY Neves Mourao E Lessa de Souza RT Da Luz Lacerda R Sibajev N Tsouris P Póvoa MM Momen H Rosa-Freitas MG Biting Indices, Host-seeking Activity and Natural Infection Rates of Anopheline species in Boa Vista, Roraima, Brazil, from 1996-1998 Mem Inst Oswaldo Cruz 2002 97 151 161 10.1590/S0074-02762002000200002 12016435

[B72] Taylor LH Infection rates in, and the number of *Plasmodium falciparum *genotypes carried by *Anopheles *mosquitoes in Tanzania Ann Trop Med Parasit 1999 93 659 662 10.1080/00034989958168 10707111

[B73] Gillies MT Wilkes TJ A study of the age-composition of populations of *Anopheles gambiae *Giles and *Anopheles funestus *Giles in North-eastern Tanzania Bull Ent Res 1965 56 237 262 10.1017/S0007485300056339 5854754

[B74] Charlwood JD Wilkes TH Studies on the age-composition of samples of *Anopheles darlingi *Root (Diptera: Culicidae) in Brazil Bull Entomol Res 1979 69 337 342 10.1017/S0007485300017818

[B75] Roberts DR Alecrim WD Tavares AM McNeill KM Field observations on the gonotrophic cycle of *Anopheles darlingi *(Diptera: Culicidae) J Med Entomol 1983 20 189 192 6842526 10.1093/jmedent/20.2.189

[B76] Tersian LA Stahler N The effect of larval population density on some laboratory characteristics of *Anopheles quadriannulatus *Say J Parasitol 1949 35 487 495 10.2307/3273653 18138183

[B77] Charlwood JD Pinto J Sousa CA Ferreira C Gil v Do Rosario VE Mating does not affect the biting behavior of Anopheles gambiae from the Islands of Sao Tomé and Principe, West Africa Ann Trop Med Parasit 2003 97 751 756 10.1179/000349803225002345 14613634

[B78] Takken W Costantini C Dolo G Hassanali A Sagnon F Osir E Mosquito Mating Behaviour http://www.library.wur.nl/frontis/disease_vectors/17_takken.pdf

[B79] Lounibos LP Lima DC Lourenço-de-Oliveira R Escher RL Nishimura N Egg maturation in neotropical malaria vectors: one blood meal is usually enough J Vector Ecology 1998 23 195 201 9879075

[B80] Monteiro de Barros FS Honório NA Man biting rate seasonal variation of malaria vectors in Roraima, Brazil Mem Inst Oswaldo Cruz 2007 102 299 302 10.1590/S0074-02762007005000024 17568934

[B81] Detinova TS Age-grouping methods in Diptera of medical importance with special reference to some vectors of malaria WHO Monogr 1962 47 1 216 13885800

[B82] Hoc TQ Charlwood JD Age determination of *Aedes cantans *using the ovarian oil injection technique Med Vet Entomol 1990 4 227 233 10.1111/j.1365-2915.1990.tb00281.x 2132986

[B83] Monteiro de Barros FS Arruda ME Vasconcelos SD Luitgards-Moura JF Confalonieri U Rosa-Freitas MG Tsouris P Lima-Camara TN Honório NA Parity and age composition for *Anopheles darlingi *Root (Diptera: Culicidae) and *Anopheles albitarsis *Lynch-Arribálzaga (Diptera: Culicidae) of the northern Amazon Basin, Brazil J Vector Ecology 2007 32 54 68 10.3376/1081-1710(2007)32[54:PAACFA]2.0.CO;2 17633426

[B84] Tadei WP Dutary-Thatcher B Malaria vectors in the Brazilian Amazon: *Anopheles *of the subgenus Nyssorhynchus Rev Inst Med Trop São Paulo 2000 42 87 94 10.1590/S0036-46652000000200005 10810323

[B85] Ulloa A Rodriguez MH Rodriguez AD Roberts DR Comparison of two collection methods for estimating abundance and parity of *Anopheles albimanus *in breeding sites and villages of southern Mexico J Am Mosq Control Assoc 1997 13 238 244 9383764

[B86] Achee NL Grieco JP Andre RG Rejmankova E Roberts DR A mark-release-recapture study using a novel portable hut design to define the flight behavior of *Anopheles darlingi *in Belize, Central America J Am Mosq Control Assoc 2005 21 366 379 10.2987/8756-971X(2006)21[366:AMSUAN]2.0.CO;2 16506561

[B87] Charlwood JW Alecrim WA Capture-recapture studies with the South American malaria vector *Anopheles darlingi*, Root Ann Trop Med Parasitol 1989 83 569 576 2619371 10.1080/00034983.1989.11812389

[B88] Takken W Charlwood JD Billingsley PF Gort G Dispersal and survival of *Anopheles funestus *and *A gambiae *s.l. (Diptera: Culicidae) during the rainy season in southeast Tanzania Bull Entomol Res 1998 88 1 6 10.1017/S0007485300041493

[B89] Zimmerman RH Ribeiro Calardo AK Lounibos LP Arruda M Wirtz R Bloodmeal Hosts of *Anopheles *species (Diptera: Culicidae) in a Malaria-endemic area of the Brazilian Amazon J Med Entomol 2006 43 947 956 10.1603/0022-2585(2006)43[947:BHOASD]2.0.CO;2 17017232

[B90] Shannon RC Anophelines of the Amazon Valley Proc Entomol Soc Washington 1933 35 117 143

[B91] Galvao ALA Damasceno RG Marques AP Algumas observações sobre a biologia dos anofelinos de importância epidemiológia em Belém Pará Arq Hig (Rio de Janeiro) 1942 12 51 11121927919

[B92] Rozendaal JA Biting and resting behavior of *Anopheles darlingi *in the Suriname rainforest J Am Mosq Control Assoc 1989 5 351 358 2584968

[B93] Pajot FX Molez JF Le Pont F *Anopheles *et paludisme sur le Haut-Oyapock (Guyane francaise) Cah ORSTOM ser Entomol Med et Parasitol 1978 16 105 111

[B94] Elliot R The influence of vector behavior on malaria transmission Am J Trop Med Hyg 1972 21 755 763 4561523 10.4269/ajtmh.1972.21.755

[B95] Lourenço-de-Oliveira R Da Gana Guimarães AE Arlé M Fernández de Silva T Conçalves Castro M Albuquerque Motta M Deane LM Anophelin species, some of their habits and relation to malaria in endemic areas od Rondõnia state, Amazon region of Brazil Mem Inst Oswaldo Cruz 1989 84 501 514 2487447 10.1590/s0074-02761989000400008

[B96] León W Valle J Naupay R Tineo E Rosas A Palomino M Comportamiento estacional del Anopheles (Nyssorhynchus) darlingi Root 1926 en localidades de Loretao y Madre de Dios, Peru 1999-2000 Rev Peru Med Exp Salud Pública 2003 20 22 27

[B97] Bustamante FM Considerações sôbre sertos problemas especiais relacionados com a erradicação da malária no Brasil Rev Bras de Malariol Doenças Trop 1959 11 9 17

[B98] Takken W Do insecticide-treated bed nets have an effect on malaria vectors? Trop Med Int Health 2002 7 1022 1030 10.1046/j.1365-3156.2002.00983.x 12460393

[B99] Guimarães AE Gentile C Lopes CM Pinto de Mello R Ecology of Mosquitoes (diptera: Culicidae) in area of Serra do Mar State Park, State of São Paulo, Brazil. III-Daily biting rhythms and lunar cycle influence Mem Inst Oswaldo Cruz 2000 95 753 760 11080757 10.1590/s0074-02762000000600002

[B100] Voorham J Intra-population plasticity of *Anopheles darlingi*'s (Diptera, Culicidae) biting activity patterns in the state of Amapá, Brazil Rev Saude Publica 2002 36 75 80 10.1590/S0034-89102002000100012 11887233

[B101] Tadei WP Thatcher BD Santos JMM Scarpassa VM Brandão Rodrigues I Rafael MS Ecological observations on Anopheline vectors of malaria in the Brazilian Amazon Am J Trop Med Hyg 1998 59 325 345 9715956 10.4269/ajtmh.1998.59.325

[B102] De Jong R Knols BGJ Bock GR, Cardew G Selection of biting sites by mosquitoes Olfaction in Mosquito-Host Interactions 1996 New York: John Wiley 89 108

[B103] Mukabana WR Takken W Coe R Knols BGJ Host-specific cues cause differential attractiveness of Kenyan men to the African malaria vector *Anopheles gambiae* Malaria Journal 2002 I 17 10.1186/1475-2875-1-17 PMC149388 12513703

[B104] Mukabana WR Takken W Killeen GF Knols BGJ Allomonal effect of breath contributes to differential attractiveness of humans to the African malaria vector *Anopheles gambiae* Malaria Journal 2004 3 1 10.1186/1475-2875-3-1 14748930 PMC343289

[B105] Knols BJG Takken W De Jong R Influence of human breath on selection of biting site by *Anopheles albimanus* J Am Mosq Control Assoc 1994 10 423 426 7807088

[B106] Hiwat H De Rijk M Andriessen R Koenraadt CJM Takken W Evaluation of Methods for Sampling the Malaria Vector *Anopheles darlingi *(Diptera, Culicidae) in Suriname and the Relation with its Biting Behavior J Med Entomol in press 10.1603/me10245 21936323

[B107] Jirakanjanakit N Leemingsawat S Thongrungkiat S Apiwathnasorn C Singhaniyom S Bellec C Dujardin JP Influence of larval density or food variation on the geometry of the wing of Aedes (Stegomyia) aegypti Trop Med Int Health 2007 12 1354 1360 10.1111/j.1365-3156.2007.01919.x 18045262

[B108] Harbach RE Roberts DR Manguin S Variation in the hindtarsal markings of *Anopheles darlingi *(Diptera; Culicidae) in Belize Mosquito Systematics 1993 25 192 197

[B109] Rosa-Freitas MG Lourenço-de-Oliveira R De Carvalho-Pinto CJ Flores-Mendoza C Silva-do-Nascimento TF Anopheline Species Complexes in Brazil. Current Knowledge of Those Related to Malaria Transmission Memórias do Instituto Oswaldo Cruz 1998 93 651 655 10.1590/S0074-02761998000500016 9830533

[B110] Mirabello L Conn J Molecular population genetics of the malaria vector Anopheles darlingi in Central and South America Heredity 2006 96 311 321 10.1038/sj.hdy.6800805 16508661

[B111] Conn JE Vineis JH Bollback JP Onyabe DY Wilkerson RC Póvoa MM Population structure of the malaria vector *Anopheles darlingi *in a malaria-endemic region of eastern Amazonian Brazil Am J Trop Med Hyg 2006 74 798 806 16687683

[B112] Mirabello L Vineis JH Yanoviak SP Scarpassa VM Póvoa MM Padilla N Achee NL Conn JE Microsatellite data suggest significant population structure and differentiation within the malaria vector Anopheles darlingi in Central and South America. BMC Ecology 2008 8 3 10.1186/1472-6785-8-3 PMC2292152 18366795

[B113] Scarpassa VM Conn JE Population genetic structure of the major malaria vector *Anopheles darlingi *(Diptera: Culicidae) from the Brazilian Amazon, using microsatellite markers Mem Inst Oswaldo Cruz 2007 102 319 27 10.1590/S0074-02762007005000045 17568937

[B114] Gutiérrez LA Gómez GF González JJ Castro MI Luckhart S Conn JE Correa MM Microgeographic genetic variation of the malaria vector *Anopheles darlingi *Root (Diptera: Culicidae) from Cordoba and Antioquia, Colombia Am J Trop Med Hyg 2010 83 38 47 20595475 10.4269/ajtmh.2010.09-0381 PMC2912573

[B115] Giglioli G Ecological change as a factor in renewed malaria transmission in an eradication area; a localized outbreak of *An. aquasalis*-transmitted malaria on the Demerara River estuary, British Guiana, in the fifteenth year of *An. darlingi *and Malaria eradication Bulletin of the World Health Organization 1963 29 131 145 14056265 PMC2554849

[B116] Takken W De Tarso R Vilarinhos P Schneider P Dos Santos F Bogers RJ, Martens P, Takken W Effects of environmental change on malaria in the Amazon region of Brazil Environmental Change and Malaria Risk Global and Local Implications 2005 11 Wageningen University Research Centre 113 123 http://library.wur.nl/frontis/environmental_change/11_takken.pdf

[B117] Patz JA Olson SH Malaria risk and temperature: Influences from global climate change and local land use practices PNAS 2006 103 5635 5636 10.1073/pnas.0601493103 16595623 PMC1458623

[B118] Vittor AY Deforestation and Malaria: Associations Between Vegetation, Vector Ecology, and Malaria Epidemiology in the Peruvian Amazon Dissertation, The Johns Hopkins University 2003 480

[B119] Vittor AY Gilman RH Tielsch J Glass G Sgields T Sánchez Lozano W Pinedo-Cancino V Patz JA The effect of deforestation on the human biting rate of Anopheles darlingi, the primary vector of falciparum malaria in the Peruvian Amazone Am J Trop Med Hyg 2006 74 3 11 16407338

[B120] Harris AF Matias-Arnez A Hill N Biting time of *Anopheles darlingi *in the Bolivian Amazon and implications for control of malaria Trans R Soc Trop Med Hyg 2006 100 45 47 10.1016/j.trstmh.2005.07.001 16154607

[B121] Povoa MM Conn JE Schlichting CD Amaral JCOF Segura MNO Da Silva ANM Dos Santos CCB Lacerde RNL De Souza RTL Galiza D Santa Rosa EP Wirtz RA Malaria Vectors, Epidemiology, and the Re-Emergence of *Anopheles darlingi *in Belem, Para, Brazil J Med Entomol 2003 40 379 386 10.1603/0022-2585-40.4.379 14680100

[B122] De Carvallo A Da influencia das variaçoes de nivel d' agua e do DDT sobre a produçao do *Anopheles darlingi* Trabalho apresentado ao XI Congresso Brasiliero de Higiene, Curitiba; Arquivos De Higiene E Saude Publica 1953

[B123] Van Thiel PH Malaria problems arising from the construction of a reservoir in the interior of Surinam Trop Geogr Med 1962 14 259 278 13993639

[B124] Zeilhofer P Dos Santos ES Ribeiro ALM Miyazaki RD Dos Santos MA Habitat suitability mapping of *Anopheles darlingi *in the surroundings of the Manso hydropower plant reservoir, Mato Grosso, Central Brazil International Journal of Health Geographics 2007 6 7 10.1186/1476-072X-6-7 17343728 PMC1851006

[B125] Silbergeld EK Nash D Trevant C Strickland GT De Souza JM Da Silva RSU Mercury exposure and malaria prevalence among gold miners in Pará, Brazil Rev Soc Bras Med Trop 2002 35 421 429 10.1590/S0037-86822002000500001 12621659

[B126] Rambajan I Reappearance of unprecedented falciparum malaria: 28 years after the last case in the Cuyuni-Mazaruni-Potaro, Guyana, South America Trop Geogr Med 1988 40 269 271 3055569

[B127] Duarte EC Fontes CJ Association between reported annual gold mining extraction and incidence of malaria in Mato Grosso-Brazil, 1985-1996 Rev Soc Bras Med Trop 2002 35 665 668 10.1590/S0037-86822002000600020 12612752

[B128] Brochero HL Rey G Buitrago LS Olano VA Biting activity and breeding sites of *Anopheles *species in the municipality villavicencio, Meta, Colombia J Am Mosq Control Assoc 2005 21 182 186 10.2987/8756-971X(2005)21[182:BAABSO]2.0.CO;2 16033120

[B129] Turell MJ Sardelis MR Jones JW Watts DM Fernandez R Carbajal F Pecor JE Klein TA Seasonal distribution, biology, and human attraction patterns of mosquitoes (Diptera: Culicidae) in a rural village and adjacent forested site near Iquitos, Peru J Med Entomol 2008 45 1165 1172 10.1603/0022-2585(2008)45[1165:SDBAHA]2.0.CO;2 19058644

[B130] Dusfour I Carinci R Gaborit P Issaly J Girod R Evaluation of four methods for collecting malaria vectors in French Guiana J Eco Entomol 2010 103 973 976 10.1603/EC09328 20568645

[B131] Verhulst NO Mukabana WR Takken W Smallegange RC Human skin microbiota and their volatiles as odour baits for the malaria mosquito *Anopheles gambiae *s.s Entomologia Experimentalis et Applicata 2011 139 170 179 10.1111/j.1570-7458.2011.01119.x

[B132] Rozendaal JA Van Hoof JP Voorham J Oostburg BF Behavioral responses of *Anopheles darlingi *in Surinam to DDT residues on house walls J Am Mosq Control Assoc 1989 5 339 350 2584967

[B133] Suarez MF Quiñones ML Palacios JD Carrillo A First record of DDT resistance in *Anopheles darlingi* J Am Mosq Control Assoc 1990 6 72 74 2324727

[B134] Santos JB Dos Santos F Macêdo V Variation of anopheles density with deltamethrin-impregnated mosquito nets in an endemic malaria area of the Brazilian Amazon Cad Saúde Pública 1999 15 281 292 10409781 10.1590/s0102-311x1999000200013

[B135] Pates H Curtis C Mosquito behavior and vector control Annual Review of Entomology 2005 50 53 70 10.1146/annurev.ento.50.071803.130439 15355233

[B136] Ferguson HM Dornhaus A Beeche A Borgemeister C Gottlieb M Mulla MS Gmnig JE Fish D Killeen GF Ecology: A prerequisite for malaria elimination and eradication PLoS Med 2010 7 8 e1000303 10.1371/journal.pmed.1000303 20689800 PMC2914634

[B137] Magris M Rubio-Palis Y Alexander N Ruiz B Galván N Frias D Blanco M Lines J Community-randomized trial of lambdacyhalothrin-treated hammock nets for malaria control in Yanomami communities in the Amazon region of Venezuela Trop Med Int Health 2007 12 392 403 10.1111/j.1365-3156.2006.01801.x 17313511

[B138] Killeen GF Fillinger U Knols BGJ Advantages of larval control for African malaria vectors: Low mobility and behavioral responsiveness of immature mosquito stages allow high effective coverage Malaria Journal 2002 1 8 10.1186/1475-2875-1-8 12153709 PMC117646

[B139] Curtis CF Radcliffe EB, Hutchison WD Control of Malaria Vectors in Africa and Asia Radcliffe's IPM World Textbook 2006 University of Minnesota, St. Paul, MN http://ipmworld.umn.edu21925821

[B140] Fillinger U Knols BGJ Becker N Efficacy and efficiency of new *Bacillus thuringiensis var. israelensis *and *Bacillus sphaericus *formulations against Afrotropical anophelines in Western Kenya Tropical Medicine and International Health 2003 8 37 47 10.1046/j.1365-3156.2003.00979.x 12535249

[B141] Kroeger A Horstick O Riedl C Kaiser A Becker N The potential for malaria control with the biological larvicide *Bacillus thuringiensis israelensis *(Bti) in Peru and Ecuador Acta Trop 1995 60 47 57 10.1016/0001-706X(95)00101-J 8546038

[B142] Nagm L Luitgards-Moura JF Neucamp CD Monteiro-de-Barros FS Honorio NA Tsouris P Rosa-Freitas MG Affinity and diversity indices for anopheline immature forms Rev Inst Med Trop Sao Paulo 2007 49 309 316 18026638 10.1590/s0036-46652007000500007

[B143] Hayes J Charlwood JD Dinamica estacional de una populacao de Anopheles darlingi, numa area endemic de malaria no Amazonas Acta Amazonica 1979 9 79 86

[B144] Davis NC A note on the malaria-carrying anophelines in Belém, Pará, and in Natal, Rio Grande do Norte, Brazil Riv Malar 1931 10 43 51

[B145] Moreno JE Rbio-Palis Y Avecedo P Indentificación de criaderos de anofelinos en un área endémica del estado Bolivar, Venezuela Bo Malariol San Amb 2000 40 21 30

